# Barriers and facilitators of using mobile devices as an educational tool by nursing students: a qualitative research

**DOI:** 10.1186/s12912-021-00750-9

**Published:** 2021-11-10

**Authors:** Nasrin Nikpeyma, Mitra Zolfaghari, Aeen Mohammadi

**Affiliations:** 1 Department of Community Health and Geriatric Nursing, School of Nursing and Midwifery, Tehran University of Medical Sciences, Tehran, Iran; 2 Department of eLearning in medical education, Virtual School of Tehran University of Medical Sciences, Naderi Street, Keshavarz Blvd, Tehran, Iran

**Keywords:** Mobile devices, E-learning, Nursing students

## Abstract

**Background:**

Although the use of mobile devices can facilitate the learning process, there may be barriers to using them for learning purposes. This study aimed to identify and investigate the barriers and facilitators of using mobile devices as an educational device from the perspective of nursing students.

**Methods:**

This qualitative descriptive study was conducted in 2020 on undergraduate nursing students of the Nursing and Midwifery Faculty, Tehran University of Medical Sciences. A total of 22 undergraduate nursing students were selected by purposive sampling with maximum variability. Inclusion criteria were Undergraduate nursing education, having mobile devices, and willingness to participate in research. Data were collected through semi-structured individual interviews for 45–75 min in the proposed environment of students until the data were saturated. Data analysis was performed manually using the framework analysis method with the steps: familiarization, identifying a thematic framework, indexing, Charting and Synthesis, Mapping, and Interpretation. Trustworthiness was determined by methods of Credibility, Dependability, Conformability, and Transferability.

**Results:**

The majority of participants (45.45%) were 21 years old, 63.63% were women, and 36.36% studied in the 8th semester. Findings from the analysis of interviews showed that barriers to the use of mobile devices were classified into 4 main categories (barriers related to mobile devices, barriers related to Internet access, barriers related to information literacy, cultural-environmental barriers) and 15 subcategories, and facilitating the use of mobile devices was divided into 2 main categories (easy to use mobile devices and easy access to information) and 6 subcategories.

**Conclusion:**

The results of this study help educational managers and curriculum planners to adapt to technological change, to focus on the many benefits of mobile devices as an educational tool, and to plan to overcome barriers to mobile device use, and use mobile devices to teach theoretical topics and clinical nursing skills effectively.

**Supplementary Information:**

The online version contains supplementary material available at 10.1186/s12912-021-00750-9.

## Background

E-learning is a process in which information and communication technologies are used to manage, design, present, select, exchange, guide, support, and develop learning. All the efforts of twenty-first-century universities are to be able to adapt to the waves of change, especially in the field of information technology. e-learning, by focusing on human beings as active learners, can transform all forms of teaching and learning [1].

Mobile learning is a new stage in the development of e-learning and distance learning. Mobile learning refers to any learning that is done through wireless mobile devices such as smartphones, mobile devices, and PCs and makes learning possible at any time and place. Although there are similarities between e-learning and mobile learning, mobile learning is somewhere beyond e-learning [2].

The latest information needed in clinical work is usually available online but rarely is there a computer or laptop in the patient’s bed. Therefore, there is a need for a mobile and practical device to access the correct information [3]. A mobile device is a small pocket and portable device whose functions, features, and capabilities are almost like a personal computer. Basic features of most mobile devices include storage for information, camera, notebook, calendar, calculator, word processor, wireless access to the Internet, and internal information networks. The operating systems of most mobile devices support many applications [4, 5].

Mobile devices are increasingly used both in nursing education and clinical practice as a method of timely access to information and resources [5, 6]. These efficient, flexible, and powerful tools can facilitate access to information and improve learning, increase efficiency, and save time [7]. Other benefits include reducing clinical errors and improving patient care [8]. The use of mobile devices helps to improve the clinical skills of nursing students, as part of nursing education. The use of these tools is based on student-centered philosophy and encourages students to think and be independent in learning [9]. Mobile apps are used to access medication information, provide clinical reports, receive peer support, and communicate between faculty and students. Access to clinical guidelines, medication resources, videos, and podcasts can improve patient safety. In a study of undergraduate nursing students, it was found that they use mobile devices as useful tools in providing patient care and 80% of clinical nurses use smartphone applications to obtain medication information [10]. Koohestani et al. (2019) reported that health students consider mobile devices as tools to facilitate learning, take steps in different learning paths, promote scientific confidence and self-management of learning, that motivate learning, promote theoretical knowledge and acquire clinical skills [11]. Despite the advantages of these devices in learning, there are some limitations for them, including practical and social limitations of these devices, such as poor access to the Internet in the clinical environment and lack of acceptance by patients and staff. Other obstacles such as lack of technical support, inability to keep the information confidential, and not having a mobile, damage or theft of the mobile were mentioned [12]. Also, the quality of scientific software, lack of memory, and small screen for mobile devices should not be overlooked [8]. Research findings on the use of mobile devices by Ontario nurses have shown that nurses do not seek the information needed for clinical decisions from credible sources. Therefore, designing appropriate systems to provide the necessary information on time can improve the performance of evidence-based nursing in such environments, and the mobile device has this ability [13].

Due to the advancement of technology and the use of new educational tools, it seems that the education of nursing students is also affected by this. Mobile devices can affect learning process the learning process of nursing students in some situations, it may present students with challenges that prevent them from learning. This study aims to investigate the barriers and facilitators of using mobile devices as an educational tool from the perspective of nursing students.

## Methods

This qualitative descriptive study was conducted in 2020 in Tehran. The study population was undergraduate nursing students of Tehran University of Medical Sciences. The inclusion criteria of this study were undergraduate nursing students, and having a mobile device, and using it as an educational tool. Participants were selected by purposive sampling with maximum variation; variable grades (low, medium, high), different semesters, and of both sexes (male and female). Sampling was continued until data saturation, and 22 undergraduate nursing students participated in this study (Table 1). Two tools were used to collect data: a) demographic information questionnaire (age, sex, semester, and grade point average) and b) semi-structured individual interview questionnaire. In semi-structured individual interviews, data were collected using the interview guide. Semi-structured interview questions were formulated according to the purpose of the research and with the general question: “What is your opinion about using mobile devices as an educational tool?” And as the interview progresses, more questions will be asked about the topic under discussion, such as: “What do you think about using mobile devices as an educational tool?”, “What do you think are the barriers/problems to using mobile devices?” “What conditions facilitate the use of mobile devices?” was asked. The interviews were conducted by the researcher.

**Table 1:** Frequency distribution of characteristics of participants

Characteristics of participants	Number	Percentage
Age		
20 years	5	22.72
21 years	10	45.45
22 years	7	31.81
Gender		
Female	14	63.64
Male	8	36.36
Semester		
Semester 4	3	13.63
Semester 5	2	9.09
Semester 6	3	13.63
Semester 7	6	27.27
Semester 8	8	36.36
Grade point average		
14–16	7	31.81
16–18	9	40.90
18–20	6	27.27
Total number	22	100

After obtaining permission to conduct research, eligible samples were selected from the list of undergraduate nursing students. The objectives of the research were explained to them and it was ensured that participation in the research was voluntary and that their information would remain confidential. After obtaining written informed consent, interviews were conducted in the place suggested by the participants (campus, classroom, internship environment). The interviews lasted 45–75 min. To record the data obtained from the interview, a digital voice recorder, and field notes were used to record participants’ non-verbal communication and interactions.

At the end of each interview, the recorded information was transferred to the paper after listening 2–3 times in the shortest possible time. Also, after the end of each session, the quality of the field notes was assessed. This process was the same for all interviews. To ensure the accuracy of the information, all the information transferred on paper was reviewed while listening to the recorded sounds. Framework analysis was used to analyze the data [14]. To perform the analysis, the following steps were performed:

1. Familiarization: To conduct this stage of the present study and to achieve a general view and immerse in the data, the recorded interviews were heard several times and the manuscripts and notes in the field were studied. The practice of repetition in reading manuscripts or listening to interviews helped to become aware of key ideas and repetitive concepts.
2. Identifying a thematic framework: In this stage of analysis, after repeated study and review of data, concepts and content that had similar meanings or were related to each other were grouped. For example, issues such as ease of transportation, access to up-to-date information, comprehensive information, and uncertainty about the validity of scientific content on sites were among the themes that emerged after repeated readings of the manuscripts.
3. Indexing: To index the data of this study, the manuscripts obtained from the interview, which were classified into different topics, were read and coded over and over again. This step was done manually.
4. Charting: After encoding the data obtained from the research, those codes that were assigned to a single topic were determined and placed in a table for easy access to all data.
5. Synthesis or Mapping and Interpretation: To combine the data of this study, all the definitions and concepts proposed by the participants were assessed, the relationship between them was determined, and finally, the barriers and facilitators of using mobile devices as an educational tool were clarified.

The trustworthiness of data was determined by Credibility, Dependability, Conformability, Transferability [15]. The credibility of the data of this study was examined through continuous engagement with the data, long-term relationships with some participants in the meetings, and checking the findings with them. In addition, the data were reviewed by other colleagues of the research team. To increase the credibility of the data, the participants were selected from among the students studying in different semesters and the variation of the semester grade point average. To assess the Dependability of the data, the information obtained from individual interviews and field notes were carefully read by at least one member of the research team, and the results were compared. To increase the Conformability of the data, all the information provided by the participants was fully presented. To transfer the data of this study, a rich and complete description of the data and context was provided.

All methods were carried out by relevant guidelines and regulations.

### Findings

**Fig. 1:**
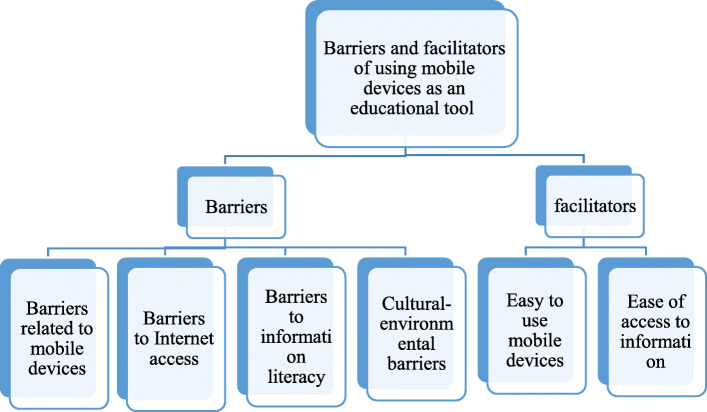
Concept map of barriers and facilitators of using mobile devices as an educational tool

Analysis of the data obtained from interviews with undergraduate nursing students about barriers and facilitators of using mobile devices as an educational tool was presented in two parts: A) Barriers to the use of mobile devices was categorized into 4 main categories (barriers related to mobile devices, barriers related to Internet access, barriers related to information literacy, cultural-environmental barriers) and 15 sub-categories, and B) Factors facilitating the use of mobile devices was categorized in 2 main categories (easy to use of mobile devices and Ease of access to information) and 6 subcategories. (Figure 1), which are described below. Each of the related classes and subcategories is discussed:

A) Barriers to using mobile devices:

1. Barriers related to mobile devices: there are barriers to using mobile devices that may make it difficult to use the device or access information. Subcategories were: filling the device memory, need for an android operating system, fewer features than a computer, and expensive mobile devices.

- 1–1 Filling the device memory: To store various scientific content, mobile devices must have adequate memory so that more content can be stored on them over time. The device memory may be full or some content may be deleted due to the storage of content or the installation of various programs and applications. Sometimes, due to the full memory of the device, it is not possible to download some content. A participant said:

“*My device memory is not large and sometimes it is not possible to download essential content, and sometimes when the device memory is full, there is a possibility of losing pre-saved files.”* (P. 5)

- 2–1 Need for android operating system: Mobile devices may have different operating systems. An android operating system is required to use some educational programs and applications. to access some scientific programs or applications, operating systems such as android are needed, which usually advanced smartphones have this system. A participant stated:

“*Advanced operating systems are needed to access software or more information, the type of my mobile is not very good, the more advanced the mobile is better, but it has more entertainment and takes our time to have a learning aspect.*” (P. 11)

- 3–1 Fewer features than computers: Mobile devices are a type of small pocket computer, and although they have the capabilities of computers, they have differences compared to computers that may limit their use. Mobile devices, despite their many advanced features, have shortcomings compared to computers that may be challenging to use. A participant said:

“*I prefer the computer because it is possible to use flash, it is possible to download, and the screen is bigger.*” (P. 14)

- 4–1 Expensive mobile devices: Mobile devices that have more advanced features are expensive and not all students may be able to have these devices. A participant said:

“*Providing a higher quality phone costs money, and simpler phones do not support some programs, mobiles must have a reputable brand and this is more expensive.”* (P. 17)

2. Barriers related to Internet access

High-speed Internet access is essential for accessing scientific content so that the learning process is not disrupted. This category includes Expensive internet packages and low internet speed.

- 1–2 Expensive internet packages: To access the Internet, it is necessary to buy Internet packages, which are both expensive and can be used for a short time. Spending money on internet packages was one of the obstacles raised by students. A participant said:

“*The price of internet packages is high and after a little searching, these packages run out.*” (P. 7)

- 2–2 Low internet speed: High-speed internet increases the speed of accessing articles or downloading scientific topics and can be achieved in a shorter time. Low internet speed or its disconnection reduces the speed of access to the content and is boring. A participant said:

“*Internet speed is low and disconnects, sometimes you have to spend a lot of time reading a scientific article or reading drug information*.” (P. 12)

3. Barriers to information literacy

Due to insufficient access to articles or the full text of them, or the existence of scientific articles only in English, this content may not be used, and sometimes due to the presentation of scientific content without reference, ensuring the validity and accuracy of information and content of some information sites is difficult. In this category are subcategories of uncertainty about the validity of scientific content on the Internet, lack of fluency in English to use scientific content, and lack of access to all information.

- 1–3 Uncertainty about the validity of scientific content on the Internet: It is difficult to ensure the accuracy of scientific content on some sites, especially when the content is duplicate and without reference. A participant said:

“*Some nursing education sites are without reference and their validity cannot be guaranteed*.” (P. 16)

- 2–3 Lack of fluency in English to use scientific content: Most up-to-date and valid nursing materials, whether in the form of books or articles, are in English, and if the student is not fluent in English, he/she cannot use it. In another word, fluency in English is necessary to access and use scientific content. A participant stated:

“*Sometimes we have to use a dictionary on our mobile regularly, and because speed is important to us, we refer to Persian content, and therefore we may not be able to find the answer to our questions.*” (P. 13)

- 3–3 Lack of access to all information: Full-text articles are not always available, and only parts of them may be accessible. In another word, it can only access the abstracts of up-to-date scientific articles, and using full-text articles requires a fee. A participant said:

“*Sometimes we find authoritative scientific articles but they only provide the abstract, and you have to pay for access to the full-text article*.” (P. 22)

4. Cultural-environmental barriers

The use of mobile devices may face physical and human barriers in clinical settings. The use of mobile devices can affect social interaction and face-to-face communication between students. Also, due to the attractiveness of different sites, the student may spend a lot of time on fun and non-educational activities. This category includes not allowed clinical environments to use their Internet, the possibility of contamination of mobile devices in the clinical environment, lack of a culture of accepting the use of mobile devices for scientific purposes, reducing interpersonal communication, dependence on mobile devices, and unscientific uses.

- 1–4 Not allowed clinical settings to use their Internet: clinical settings, including hospitals and medical centers, are equipped with Wi-Fi, but nursing students cannot use this possibility. A participant stated:

“*Some wards of the hospital have Wi-Fi, but do not provide the password to students, and if you do not have an Internet package, it is not possible to use the Internet*.” (P. 15)

- 2–4 Possibility of contamination of mobile device in a clinical setting: Hands may become contaminated during contact with the patient, surfaces, or equipment, and this contamination may be transmitted to the mobile device before hand washing. In clinical settings, including hospitals and medical centers, the use of the mobile device may cause contamination and this contamination is transmitted. A participant stated:

“*Contamination of the mobile prevents working with them in clinical settings. For example, I wanted to calculate the dose of the drug. My hand was so dirty, that I could not touch the phone.”* (P. 21)

- 3–4 Lack of a culture of accepting the use of mobile devices for scientific purposes: The use of mobile devices, as a means of learning, in clinical settings, has not yet been accepted by staff, instructors, and patients and maybe oppose its use. A participant said:

“*When I enter the hospital wards or at the patient’s bedside, and I have a mobile device in my hand, the staff or the patient may not realize that I am using the phone to search for scientific information or calculate the dose of medicine and they think with the phone I am entertained*.” (P. 14)

- 4–4 Reducing interpersonal communication: The use of mobile devices can negatively affect interpersonal relationships. In another word, using the mobile device to obtain information and spend time, can harm the human relationship between the student and the instructor, the student, and other students and the student with the patient and staff. Reduced social interaction is one of the disadvantages of using mobile devices. One participant explained:

“*Using these tools reduces our contact with friends and the instructor, when the scientific content is explained by the instructor is more useful both in terms of education and communi*cation.” (P. 8)

- 5–4 Dependence on the mobile device: Continuous use of the mobile device to obtain scientific information, and ensure the permanent existence of this device can affect the long-term memory of students, and can scientifically make a person dependent on the device. A participant stated:

“*Phones cause us to save information to short-term memory and forget it quickly, and this is not good. Using a mobile, although fast, is also quickly erased from our memory, and the mobile should always be with me*.” (P. 9)

- 6–4 Non-scientific uses: When using mobile devices, due to curiosity or charm of some entertainment sites, it may cause distraction and take a lot of time. The use of mobile devices may become out of scientific mode. A participant said:

“*Using mobile makes me spend a lot of time looking at non-educational and non-working sites, and sometimes I come to myself and see that I have been busy for hours without any results.”* (P. 19)

B) Factors facilitating the use of mobile devices:

1. Easy to use mobile devices

Easy-to-use mobile devices, as small computers, are conditions that make it possible to use this device everywhere. This category consists of ease of carrying mobile devices.

- 1–1 Ease of carrying mobile devices: Students introduced mobile devices as light, small and portable devices. One of the participants said:

“*These devices are so light and small that they fit easily in the pocket, and can be used in internships.”* (P. 21)

2. Ease of access to information

Ease of access to information means access to required and up-to-date information at any time and place by the user. Mobile devices such as smartphones can both access and store information so that it can be used when needed. This category has 5 sub-categories: access to up-to-date information, the possibility of updating the programs installed on the mobile device, the combination of theoretical and practical training in the clinical setting, the possibility of storing and accessing resources, and comprehensive information.

- 1–2 Access to up-to-date information: It is possible to communicate with the world by using mobile devices, and while having access to up-to-date scientific information in the field of nursing, it is possible to communicate and ask questions from scientific and expert people. One of the participants stated:

“*We can easily use up-to-date methods to learn ECG interpretation or calculate the dose of drugs. How much medicine should this patient with this condition take per minute? To make sure, I also calculated manually, the answer was the same*” (P.19)

- 2–2 Possibility to update the programs installed on the mobile device: To learn some scientific content, there are up-to-date software and applications that can be installed on mobile devices. A participant stated:

“*You can update the program or application on the mobile. You may see a newer program on another friend’s mobile and want to download that program.”* (P.1)

- 3.2 Combination of theoretical and practical training in the clinical setting: Using mobile devices, clinical skills can be combined with knowledge and scientific information related to the same skill. A participant said:

“*When I go to the patient’s bedside, I cannot take a book or a computer with me, but I can use my mobile phone and, while practicing my clinical skills, be scientifically confident*.” (P. 10)

- 4–2 Possibility of storing and accessing resources: Mobile devices can store scientific content on them. Storing information on mobile devices helps to be able to refer to them when needed or to read them at the right time to remember. A participant said:

“*Sometimes I save the PDF file of the books and when I need it, I go to the list and find the content. It would be great if they could provide a PDF of the nursing reference translated into Persian so that it can be used on the phone, and this is very good because it takes time to read English references*.” (P. 16)

- 5.2 Comprehensive information: Mobile devices can provide access to comprehensive information at any time and place. A participant stated:

“*When you want to assess the patient and take a history, although the patient file can help, to complete information about the disease and medications, you can search the valid sites and find the answer to the question.”* (P. 13)

## Discussion

The findings of this study showed that barriers to using mobile devices as an educational tool in 4 main categories: a) barriers related to mobile devices, b) barriers related to internet access, c) barriers related to information literacy, and d) cultural-environmental barriers.

### Barriers related to mobile devices

The results of this study showed that mobile devices have limitations compared to computers. Among these limitations is the lack of memory capacity of mobile devices that cannot store all the scientific, useful, and practical information, or the small size of the mobile device that prevents reading content or viewing educational images and videos. Also, to use some applications, the Android operating system is required. Strandell-Laine et al. (2015) reported the small size of the mobile device screen compared to a computer as a barrier to using these devices [16]. George et al. (2017) reported that despite the possibilities of mobile devices, due to the small size of the screen or the inability of some of these devices to use scientific content, medical students prefer computers to conduct evidence-based research [10]. Computers are not portable, but they have more capabilities and may be more useful for storing information or searching for resources. Due to the small screen and portability of mobile electronics, there are problems for the health of their users compared to computers. These problems include eye fatigue due to the small font and pressure on the neck due to improper positioning when using them.

Another barrier to the use of mobile devices was the high cost of these devices, which were not available to all students, especially the advanced type. Different results of studies have been expressed about the price of mobile devices. Mobile device such as smartphones have been popular among higher education students for various reasons, such as being cheaper than pocket computers [2]. Initially, buying a mobile device is expensive, but due to its useful applications as an educational tool, it is cost-effective. In another study, lack of access to mobile devices due to their high cost was one of the obstacles to the development of e-learning [17]. Mobile devices are more vulnerable because they may fall and break, and while it takes time to repurchase, a lot of money has to be paid.

### Barriers to internet access

The results of this study showed that the high cost of internet packages and low speed of internet are barriers to using mobile devices as an educational tool. Internet packages cost a lot, which is a high cost for a student, and because the internet speed is low, the search for scientific content is slow, and after a little use, the internet package runs out. In some studies, problems with Internet connection in the clinical environment were mentioned as barriers to the use of mobile technology [17, 18]. O’Doherty et al. (2018) also found that one of the barriers to using mobile technology in learning is the lack of continuous access to the internet due to a lack of financial resources [17]. Lack of internet speed to search for scientific materials causes fatigue, while interrupting the search may reduce the student’s motivation to continue the search.

### Barriers to information literacy

The results of this study showed that participants are sometimes unsure of the validity of scientific content available on the internet or due to lack of English language proficiency, cannot use English language scientific content. Participants also stated that sometimes they could not access full-text articles and that this would cost money. In other words, students’ ability to use and evaluate scientific and valid information is low, or they cannot use the available technology well to access useful content. Lin&Lin (2016) stated that the credibility of the virtual environment is important for learning. Despite the rapid growth of information and the advancement of communication technologies, there is still a lack of a technology-based learning approach to integrate real and virtual environments to solve problems in nursing education [19]. Although e-learning and the use of mobile devices have advanced in education, the method of teaching is individual and there is still no single solution for validating or verifying information. Studies have reported that despite the effectiveness of mobile learning tools in increasing nursing students ‘knowledge and clinical skills, their satisfaction with learning based on mobile learning tools has not been high, which may be due to students’ lack of familiarity with new technologies [18, 20].

### Cultural-environmental barriers

The use of mobile devices may encounter physical and human barriers in clinical settings. Lack of permission to use the internet in clinical settings, lack of a culture of using mobile devices, and its rejection by staff for scientific purposes were barriers mentioned by the participants. Clinical settings are equipped with Wi-Fi and high-speed internet but do not provide students with a password to use it. On the other hand, the use of mobile devices by students in clinical settings may be resisted by staff, patients, or educators, and they do not view these devices as a means of teaching and learning, but as a means of escape from the responsibility of caring for the patient. Strandell-Laine et al. (2015) found that the prevailing culture in hospital wards during clinical practice includes the law prohibiting the use of mobile devices in wards, negative attitudes of staff, and hospital rules as barriers to mobile devices [16]. The culture of using mobile devices as an educational tool requires rules and regulations, and these rules must be accepted by the nursing staff. The nursing staff believes that the nursing profession is based on clinical skills and that students should be involved in providing patient care. Nursing staff still consider mobile devices as a means of entertainment or non-educational means, which diverts students from providing care to the patient. George et al. (2017) also reported that nursing students consider mobile devices as useful tools in providing patient care and clinical decision making, but nursing administrators consider the use of these devices in the patient’s bed as unprofessional and immoral [10].

Another barrier mentioned by the participants in this study was the possibility of contaminating mobile devices at the patient’s bedside. In hospital wards, the possibility of transmission, especially through the hands, is high, making it difficult to use mobile devices. Lin&Lin (2016) also found that negative perceptions of staff and patients, and issues such as difficulty in keeping patient information confidential and the possibility of infection transmission were reported as barriers to the use of mobile devices [19]. Another barrier was the reduction of interpersonal communication. The students expressed that the use of mobile devices affects the interaction with the instructor, friends, staff, and especially patients, and reduces the feeling of empathy and sympathy, and patients may feel that their needs are not taken into account or disrespected. Yahyazadeh et al. (2016) also reported that the use of mobile devices may affect nursing students ‘empathy for patients and even interfere with care, such as prescribing medications and endangering patients’ safety [21]. Patients need attention, tend to talk about their needs and problems with health care providers, and feel empathy or sympathy. The use of mobile devices as an educational tool is based on individual education and one of the disadvantages of this educational method is the reduction of interpersonal interactions and sometimes social isolation.

Mobile device dependence and unscientific uses were other barriers expressed by participants. Mobile device dependence means that it is necessary to answer scientific or non-scientific questions or to find appropriate solutions, and this may reduce the power of thinking and analysis or the use of individual memory, and even Influence people to study and search. Using mobile devices sometimes leads to searching on unscientific and fun sites, and this is both time-consuming and an obstacle to achieving learning goals. Kahyaoglu Sut, et al. (2016) also reported that excessive use of mobile devices may be a form of entertainment and spending time on social networks and cause disorders in students’ mental health, so that their social relationships are reduced and cause social isolation, loneliness and even become depressed [22]. Sometimes dependence on mobile devices causes addiction for the student and the distance of these devices can cause a feeling of social anxiety in the student.

In explaining the opinions of nursing students, facilitators of using mobile devices as an educational tool are divided into 2 main categories: a) easy use of mobile devices and b) ease of access to information.

### Easy use of mobile device

The easy use of mobile devices meant that these devices, in addition to having the facilities of a computer, are small and portable and can be used everywhere. In nursing education, the use of mobile devices goes beyond storing and organizing information or wireless access to the internet and is used for clinical reasoning to solve problems and clinical decisions [4]. Students’ learning needs are different and mobile devices, as student-centered learning tools, can meet the different needs of students in a variety of learning situations. HeeKim & Park (2019) also stated that the use of mobile devices promotes active learning in students and promotes cooperation and communication between students and due to their small size and lightness, these devices are easily used for access to social media and scientific communication with researchers and experts [20].

### Ease of access to information

The results of this study showed that mobile devices facilitate access to information and make it possible to combine theoretical training and clinical skills. In other words, students can access credible resources at any time and find appropriate answers to their educational or clinical questions (such as drug side effects, nursing care). They can also answer questions related to clinical skills and patient care while performing procedures or clinical skills or even access instructional videos on how to do clinical skills. Strandell-Laine et al. (2015) also found that mobile devices can help nursing students make decisions, access information, and facilitate learning in clinical education [16]. Access to up-to-date and scientific information helps the student to analyze, critique or prioritize information and can meet the cognitive goals of education. Also, the implementation of innovative methods of nursing education provides the learner to move without time and space limitations, because nursing education is a combination of conceptual knowledge and practical skills in different places such as classrooms, laboratories, and clinical wards, and nursing students can access information [11, 23].

The results of this study showed that one of the factors facilitating the use of mobile devices as an educational tool is the possibility to update the programs installed on the mobile device. This means that some nursing education applications or care plans and clinical guidelines are updated, and students can replace these new programs with previous programs on their mobile devices and access new and up-to-date information. George et al. (2017) reported that students pay more attention to mobile learning tools than reference books because the instructions are constantly updated and their applications are often free. The use of applications in the nursing education program leads to active learning and the provision of evidence-based nursing care [10]. Kim&Suh (2018) reported that the use of nursing skills applications on mobile devices is effective on knowledge, self-efficacy, and implementation of nursing students’ skills [9]. Because the nursing profession is a theoretical-practical profession, so knowledge and skills are combined, and mobile devices as educational tools can increase people’s knowledge and by simulating or nursing skills videos, strengthen students’ skills.

The results of this study showed that one of the factors facilitating the use of mobile devices by students has been the possibility of storing and accessing comprehensive resources and information. This means that students can store up-to-date scientific information on their mobile devices and use it if needed. In addition, in the case of an educational subject, they can access various authoritative sources and obtain comprehensive information about a scientific subject. Chase et al. (2018) also stated that mobile devices provide access to a wide range of resources, including anatomy, medication information, clinical scoring systems, and e-books for medical students. Mobile devices, like traditional education, are effective both in clinical settings and informal educational settings and increase the speed and ease of access to information [12]. Also, the use of mobile devices, as an educational tool in nursing, can facilitate access to resources for teachers and learners and provide communication between the real and virtual worlds [19].

One of the limitations of qualitative research is the generalizability of the findings, which is no exception to this research, while this research is based on the cultural context of Iran. According to the findings of this study, it is suggested that solutions remove barriers to the use of mobile devices as an educational tool from the perspective of nursing students and teachers. Also, strategies to promote the use of mobile devices as an educational tool in theoretical and practical nursing courses to be examined.

## Conclusion

The results of this study showed that mobile devices are used as an educational tool by nursing students and are effective in increasing their knowledge and learning clinical skills. In this study, the most important barriers mentioned in using mobile devices as an educational tool included: barriers related to mobile devices, barriers related to Internet access, barriers related to information literacy and cultural-environmental barriers, and the most important facilitating factors The user included easy access to information and easy use of mobile devices. The results of this study help educational administrators and curriculum planners in the field of nursing to take action to remove the barriers by considering the benefits of mobile devices and pay more attention to use mobile devices to develop e-learning in theoretical courses and clinical skills of nursing students.

## Supplementary Information

**Additional file 1.**

## Data Availability

The datasets used and/or analyzed during the current study are available from the corresponding author on request.
