# Supplementary material for: Barriers and facilitators of using mobile devices as an educational tool by nursing students: a qualitative research
Source: BMC Nurs. 2021 Nov 10;20:226. doi: 10.1186/s12912-021-00750-9 (PMC8579623; doi:10.1186/s12912-021-00750-9)
Supplement: Supplementary file 1 — Additional file 1. [file 12912_2021_750_MOESM1_ESM.docx]

**Questionnaire:**

1. **Demographic information**

Age: ………………

Sex: Female Male

Semester: ……………..

Grade point average: …………………

1. **Semi structured questions:**

- What is your opinion about using mobile devices as an educational tool?
- What do you think about using mobile devices as an educational tool?
- What do you think are the barriers/problems to using mobile devices?
- What conditions prevent access to scientific information when using a mobile device as an educational tool?
- What conditions facilitate the use of mobile devices?
- When using a mobile device as an educational tool, what conditions make it easier to access scientific information?
- What are the capabilities of a mobile device as an educational device?
- Describe your experience of using a mobile device as an educational tool in a day?
